# Empagliflozin and Dulaglutide are Effective against Obesity-induced Airway Hyperresponsiveness and Fibrosis in A Murine Model

**DOI:** 10.1038/s41598-019-51648-1

**Published:** 2019-10-30

**Authors:** Hye Jung Park, Heejae Han, Eun-Yi Oh, Sung Ryeol Kim, Kyung Hee Park, Jae-Hyun Lee, Jung-Won Park

**Affiliations:** 1 Department of Internal Medicine, Gangnam Severance Hospital, Yonsei University College of Medicine, Seoul, Korea; 2 Institute of Allergy, Yonsei University College of Medicine, Seoul, Korea; 3 Division of Allergy and Immunology, Department of Internal Medicine, Yonsei University College of Medicine, Seoul, Korea

**Keywords:** Asthma, Preclinical research

## Abstract

Patients with asthma with obesity experience severe symptoms, are unresponsive to conventional asthma treatment, and lack proper pharmacotherapy. Empagliflozin and dulaglutide, developed for diabetes, reduce weight, decrease insulin resistance, and exert additive effects. We evaluated the efficacy of empagliflozin, dulaglutide, and their combination on obesity-induced airway hyperresponsiveness (AHR) and lung fibrosis using a murine model. We assigned C57BL/6J mice to five groups: control, high-fat diet (HFD), and HFD with empagliflozin, dulaglutide, or both. Mice received a 12-week HFD, empagliflozin (5 days/week, oral gavage), and dulaglutide (once weekly, intraperitoneally). Both drugs significantly attenuated HFD-induced weight increase, abnormal glucose metabolism, and abnormal serum levels of leptin and insulin, and co-treatment was more effective. Both drugs significantly alleviated HFD-induced AHR, increased macrophages in bronchoalveolar lavage fluid (BALF), and co-treatment was more effective on AHR. HFD-induced lung fibrosis was decreased by both drugs alone and combined. HFD induced interleukin (IL)-17, transforming growth factor (TGF)-*β*1, and IL-1*β* mRNA and protein expression, which was significantly reduced by empagliflozin, dulaglutide, and their combination. Tumour necrosis factor (TNF)-*α* and IL-6 showed similar patterns without significant differences. HFD-enhanced T helper (Th) 1 and Th17 cell differentiation was improved by both drugs. Empagliflozin and dulaglutide could be a promising therapy for obesity-induced asthma and showed additive effects in combination.

## Introduction

Obesity has been recognized as an important risk and aggravating factor for asthma^1,2^. Physical limitation on motion of lung volume, metabolic abnormality including insulin resistance, innate immunity, non-T helper (Th) 2 type inflammatory signals, and fibrosis are suggested to be main mechanisms of obesity-induced asthma development and exacerbation^3^. With the increased prevalence of obesity and asthma, obesity-induced asthma has emerged as a serious problem that needs to be addressed^1,4^. Patients with asthma who have obesity experience severe symptoms, do not respond to conventional asthma treatment including inhaled corticosteroids, and progress to a condition with a poor prognosis^5,6^. Current recommendations to treat obesity-induced asthma are mainly lifestyle modifications to lose weight and bariatric surgery to reduce energy intake^7,8^. Although some anti-obesity drugs have shown significant effects in controlling asthma in previous studies^9^, there are no drugs which are clinically used for the treatment of obesity-induced asthma. Until now, numerous researchers are currently working on a new candidate drug^10^.

Some anti-obesity drugs have emerged. Empagliflozin, a sodium/glucose cotransporter (SGLT) 2 inhibitor developed to treat diabetes, acts by inhibiting glucose reabsorption in the kidney to control glucose levels without hypoglycaemic side effects, leading to improvement of insulin resistance and weight loss^11^. Dulaglutide, a glucagon-like peptide (GLP) 1 receptor agonist, was also developed to control glucose levels. It increases signaling on insulin secretion, inhibits glucagon release when glucose levels are elevated, and delays gastric emptying, leading to weight loss^12^. The combination of an SGLT2 inhibitor and a GLP1 agonist is known to have synergic effects on glucose control and weight reduction^13^. We expected these two anti-obesity drugs to improve disease control of obesity-induced asthma. We also expected the combination of both drugs to be more effective than monotherapy.

We aimed to determine whether empagliflozin and dulaglutide alone or combined would be effective against obesity-induced airway hyperresponsiveness (AHR) and lung fibrosis using a murine model.

## Results

### Empagliflozin and dulaglutide improved high fat diet (HFD)-induced weight increase

**Figure 1:**
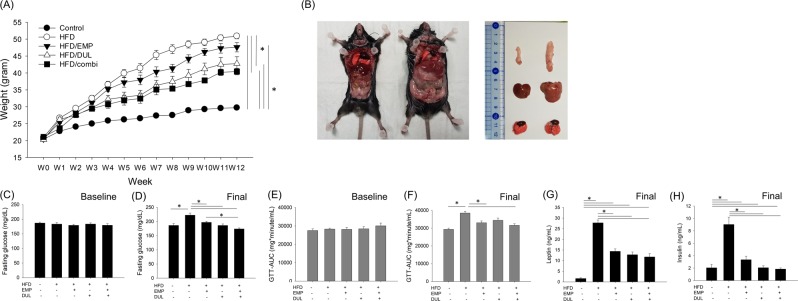
We designed 5 groups as follows: normal diet control (n = 6), HFD-induced obesity (n = 7), HFD/EMP (n = 5), HFD/DUL (n = 7), and HFD/combi (n = 7). Weight changes among groups (**A**), gross appearance and volume of internal organ in control group (left) and HFD group (right) (**B**), baseline fasting glucose level among groups at week 0 (**C**), fasting glucose level among groups at week 12 (**D**), baseline GTT-AUC among groups at week 0 (**E**), GTT-AUC among groups at week 12 (**F**), serum level of leptin (**G**) and insulin (**H**) among groups at week 12. Repeated-measures analysis of variance (ANOVA) followed by a post-hoc Bonferroni test was used in Fig. 2A and one-way ANOVA followed by a post-hoc Bonferroni test was used in Fig. 2C-F. HFD, high-fat diet; EMP, empagliflozin; DUL, dulaglutide; combi, combination of EMP and DUL; GTT, glucose tolerance test; AUC, area under the curve.

Compared to the control group fed a normal chow diet with a mean weight of 29.7 g, the HFD group showed a tremendous increase in weight at 50.9 g on the final date, which was 171.4% of the average weight of the control group (Fig. 1A). The gross appearance and volume of the organs including the lung, liver, and adipose tissue were much larger in the HFD group than they were in the control group (Fig. 1B). The final weights of the HFD-induced obesity with empagliflozin (HFD/EMP), with dulaglutide (HFD/DUL), and with a combination of empagliflozin and dulaglutide (HFD/combi) groups were 47.6, 42.8, and 40.4 g (160.2, 144.1, and 136.0% of the average weight of the control group), respectively. Although empagliflozin alone did not significantly reduce the weight increase, dulaglutide alone and combined with empagliflozin were significantly effective in reducing the weight increase (Fig. 1A).

### Empagliflozin and dulaglutide improved HFD-induced metabolic abnormality

Baseline fasting glucose and glucose tolerance test (GTT)-area under the curve (AUC) assessed at week 0 were not different among the groups; however, after 12 weeks, the values were significantly higher in the HFD group than in the control group. Empagliflozin, dulaglutide, and their combination significantly reduced fasting glucose level. In addition, co-therapy more effectively reduced the fasting glucose than empagliflozin alone did. Empagliflozin and the co-treatment reduced the GTT-AUC; however, dulaglutide was not significantly effective (Fig. 1C–F). Serum levels of leptin (Fig. 1G) and insulin (Fig. 1H) were significantly elevated in HFD group compared to that in control group, however, it was significantly reduced in all the treatment group.

### Empagliflozin and dulaglutide alleviated HFD-induced AHR and airway inflammation

**Figure 2:**
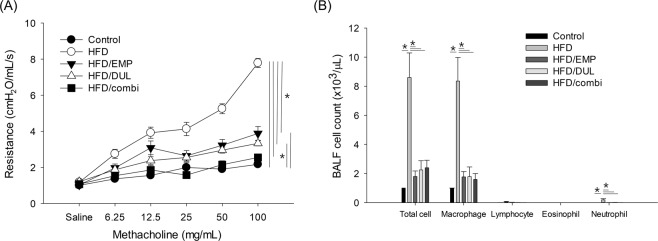
Airway hyperresponsiveness (AHR, **A**) and bronchoalveolar lavage fluid (BALF) cell count (**B**) among groups. Repeated-measures analysis of variance (ANOVA) followed by a post-hoc Bonferroni test was used in Fig. 3A and one-way ANOVA followed by a post-hoc Bonferroni test was used in Fig. 3B. HFD, high-fat diet; EMP, empagliflozin; DUL, dulaglutide; combi, combination of EMP and DUL.

Compared to the control group, the HFD group showed significant AHR. Empagliflozin, dulaglutide, and the co-treatment significantly alleviated HFD-induced AHR. However, the AHR in the HFD/EMP and HFD/DUL groups was significantly different from that in the control group. In addition, the HFD/combi group showed a further reduction in the AHR to levels comparable to that of the control group (Fig. 2A). Compared to the control group, the HFD group showed a significant increase in bronchoalveolar lavage fluid (BALF) cells with macrophage dominance. Empagliflozin, dulaglutide, and the combination significantly alleviated HFD-induced increase in BALF cells, especially macrophages and neutrophils (Fig. 2B).

### Empagliflozin and dulaglutide alleviated HFD-induced fibrosis

**Figure 3:**
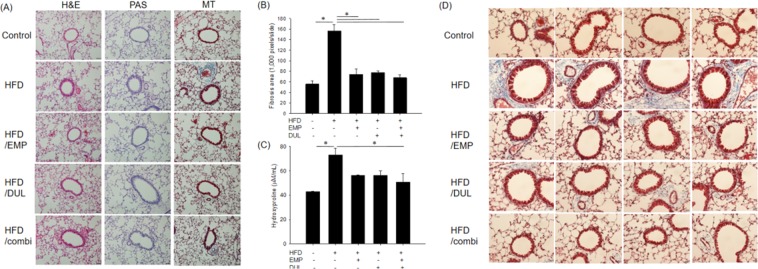
Histopathological assessment using haematoxylin and eosin (H&E), periodic acid-Schiff (PAS), and Mason’s trichrome (MT) staining (×200) (**A**), quantitative fibrosis area assessed using MT staining (**B**), and hydroxyproline level (**C**) among groups. Fibrosis was more predominantly observed in peribronchial and perivascular area compared that in lung parenchymal lesion in MT staining (×400) (**D**). One-way ANOVA followed by a post-hoc Bonferroni test was used in Fig. 4B,C. HFD, high-fat diet; EMP, empagliflozin; DUL, dulaglutide; combi, co-treatment with EMP and DUL.

The results of histopathological analysis performed using haematoxylin and eosin (H&E) and periodic acid-Schiff (PAS) staining were not different among the groups, which showed similar cellular proliferation and infiltration and goblet cell hyperplasia. However, Masson’s trichrome (MT) staining revealed that HFD induced significant fibrosis compared to the control group. Empagliflozin, dulaglutide, and the co-treatment significantly alleviated HFD-induced fibrosis (Fig. 3A). The fibrotic area in the HFD group was significantly broader than that in the control group. Empagliflozin, dulaglutide, and the co-treatment significantly alleviated the extent of HFD-induced fibrosis area to levels in the control group (Fig. 3B). Hydroxyproline level significantly increased in the HFD group compared to that in the control group and this effect was significantly improved in the HFD/combi group (Fig. 3C). Fibrosis was more predominantly observed in peribronchial and perivascular area compared that in lung parenchymal lesion (Fig. 3D).

### Empagliflozin and dulaglutide improved HFD-induced increase in cytokine mRNA expression

**Figure 4:**
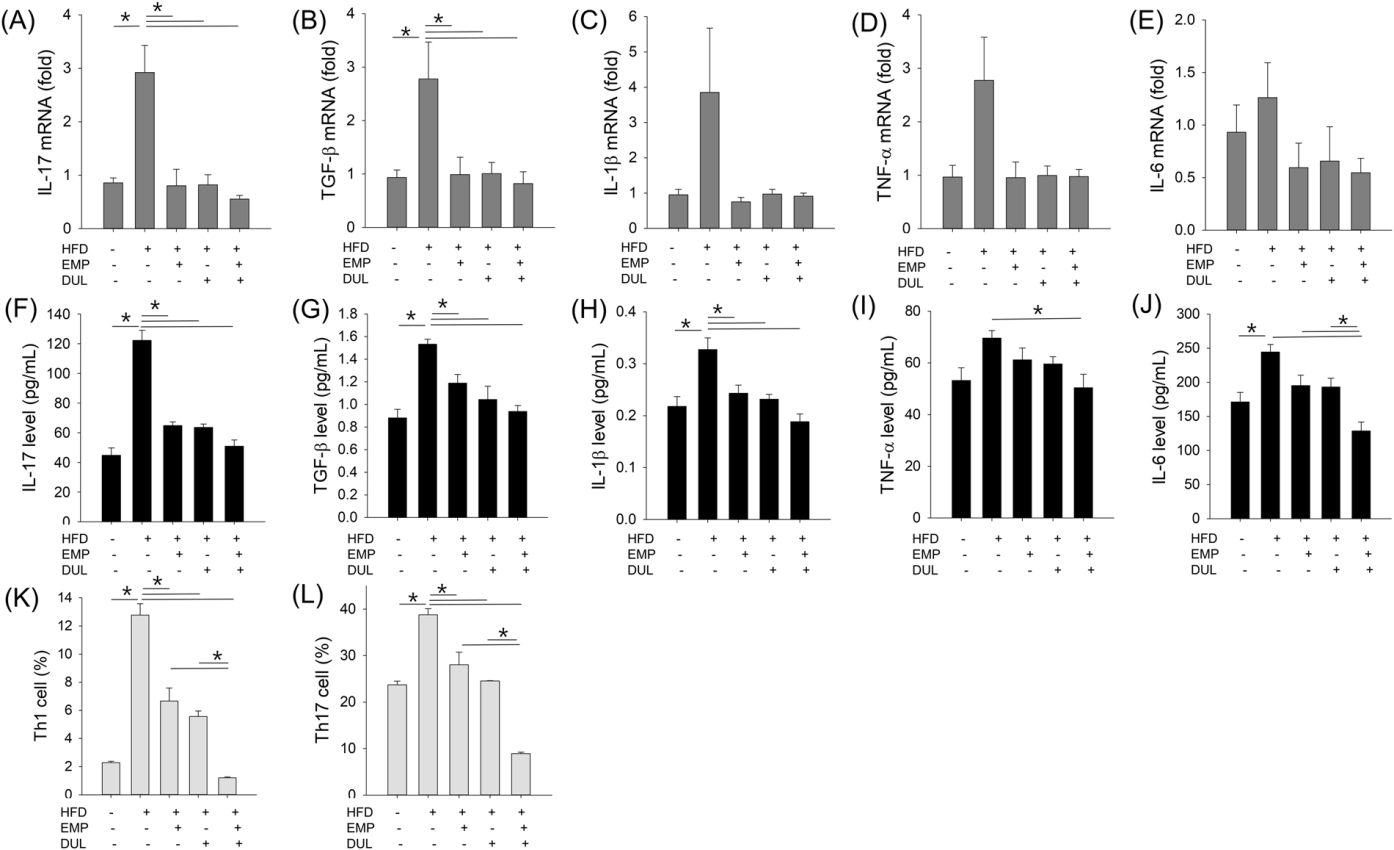
Cytokine mRNA expression (**A**–**E**), cytokine level (**F**–**J**), and Th cell differentiation (**K, L**) among groups. One-way ANOVA followed by a post-hoc Bonferroni test was used in Fig. 5. HFD, high-fat diet; EMP, empagliflozin; DUL, dulaglutide.

We assessed cytokine mRNA expression to evaluate the effects of the drugs on mRNA inflammatory signals. Interleukin (IL)-17 and transforming growth factor (TGF)-*β*1 mRNA expression significantly increased in the HFD group compared to the control group, whereas empagliflozin, dulaglutide, and the co-treatment significantly reduced these levels to those in the control group. IL-1*β*, tumour necrosis factor (TNF)-*α*, and IL-6 mRNAs showed a similar pattern; however, they were not statistically significant (Fig. 4A–E).

### Empagliflozin and dulaglutide improved HFD induced cytokine productions

Cytokine levels in lung homogenates were assessed using two site-ELISA kits to evaluate the effects of drugs on inflammatory protein levels. IL-17, TGF-*β*1, and IL-1*β* levels, which significantly increased in the HFD group compared to the control group, were significantly reduced in the HFD/EMP, HFD/DUL, and HFD/combi groups to the control group levels. TNF-*α* and IL-6 showed a similar pattern but no statistical significance was found with empagliflozin or dulaglutide. Only the co-treatment significantly reduced TNF-*α* and IL-6 levels compared to those of the HFD group. In addition, the HFD/combi group showed significantly reduced IL-6 levels compared to that in the HFD/EMP and HFD/DUL groups (Fig. 4F–J).

### Empagliflozin and dulaglutide improved HFD-induced increase in Th cell differentiation

Th cell differentiation was analysed to determine the effects of drugs on cellular inflammatory signals. HFD induced active differentiation of Th1 and Th17 cells, which was significantly decreased by empagliflozin, dulaglutide, and the co-treatment. In addition, the co-treatment showed higher effects than did empagliflozin alone and dulaglutide alone (Fig. 4K,L).

## Discussion

This is the first study to confirm that empagliflozin and dulaglutide improve AHR and fibrosis, thereby suppressing obesity-induced asthma in a murine model. Obesity alone (weight increase ≥145%) without any allergenic sensitization can induce significant asthma with non-Th2 type non-eosinophilic features^14,15^. Various mechanisms including metabolic abnormality, innate immunity, and specific cytokines have been implicated in these effects^3^. Weight-loss interventions have been first postulated as therapeutic options to improve asthma outcomes in obesity-induced asthma patients. There are emerging studies on the effects of lifestyle modification and bariatric surgery on obesity-induced asthma; however, few have investigated the effects of pharmacotherapy^16^. To date, no anti-obesity drugs are used to treat obesity-induced asthma, clinically. For the first time, we demonstrated the potential usefulness of the anti-obesity drugs, empagliflozin and dulaglutide, in treating obesity-induced asthma using an HFD-induced obesity murine model.

Empagliflozin and dulaglutide were originally developed to manage diabetes^11,12^. Then, this study showed significant improvement of glucose metabolism in both two drugs, although EMP did not reach significant weight loss. However, these drugs have additional weight-loss effects without hypoglycaemic adverse reaction. Contrary to EMP, DUL had direct effect on gastric emptying, and has more strong effects on weight loss. We also showed similar results in this study, moreover we showed additive effects on weight loss using both drugs. Only one clinical study has suggested that the anti-obesity drugs, sibutramine and orlistat, combined with dietary restriction could improve asthma control^9^. Contrary to these older anti-obesity drugs, empagliflozin and dulaglutide have no psychotropic or gastrointestinal stimulating effects; therefore, they can be used safely for a relatively prolonged period^11,12^. Therefore, we hypothesised that these drugs could be promising candidates to manage obesity-induced asthma in clinical use. We demonstrated the plausibility that these two drugs could significantly reduce weight increase, which attenuated HFD-induced inflammation, lung fibrosis, and AHR: *indirect effects*.

We also showed that co-treatment with these two drugs had additive effects on HFD-induced AHR and fibrosis. This may have been mediated by superior weight reduction of combination compared to that of either empagliflozin and dulaglutide. Additive effect of the co-treatment on weight reduction were also revealed in a previous human study^13^. Because of this effect, contrary to each monotherapy, the co-therapy completely prevented the development of AHR and fibrosis by HFD. In summary, we confirmed that this additive effect is also potentially effective in the management of obesity-induced adsthma via weight reduction.

Metabolic abnormality has been suggested as the mechanism of obesity-induced asthma. Adipokines, including adiponectin and leptin, secreted by adipose tissues are important hormones for regulating metabolic process, energy homeostasis, and inflammation. Reduced adiponectin and increased leptin level, which are frequently observed in obesity could induce AHR^17,18^. Hyperglycaemia and insulin resistance are also suggested to be risk factors for asthma and lung fibrosis^19,20^. Previous studies have showed that obesity induce impairment of insulin signaling, which can lead to bronchoconstriction, fibrosis, and AHR^21,22^. Empagliflozin and dulaglutide restored the metabolic abnormality, including leptin and insulin levels, in this study model. Both the weight loss and restorative effects on the metabolic abnormality may alleviate obesity-induced asthma.

This study showed that the weight loss effects of empagliflozin are slightly weaker than those of dulaglutide and their combination; however, empagliflozin was also significantly effective against AHR and fibrosis. Then, we can assume that weight reduction and improvement of glucose metabolism alone does not seem to contribute to this mechanism. Obesity leads to asthma via various mechanisms including inflammatory signalling and fibrotic changes; therefore, recent studies have revealed that anti-inflammatory or anti-fibrotic drugs including roflumilast, anti-IL-17 and anti-IL-1*β* antibodies could have significant effects on obesity-induced asthma without significant weight loss^10,23,24^. Empagliflozin and dulaglutide have anti-inflammatory effects by direct and indirect effects on inflammatory signalling, oxidative stress, and cytokine production^14,25–27^. In addition, the these drugs can result in anti-fibrosis effects^22^. We think the additional anti-inflammatory and anti-fibrotic effects of these drugs might enhance their positive treatment effects on obesity-induced asthma: *direct effect*.

We also showed that obesity did not induce overt inflammation in airway and lung parenchyme in this study. Many previous studies have described that obesity lead to changes of adipokines, increases of cytokine level, increases of fibrosis, however it did not lead to cellular infiltration in lung tissue^10,14,28^. This phenomenon had called ‘low-grade systemic inflammation’ or ‘pauci inflammation’^14^. This study also showed similar results to previous studies.

Obesity-induced asthma presents with severe symptoms that do not respond to conventional asthma treatment, which subsequently proceeds to poor outcomes in patients^5,6^. Currently, there is no specific drug for the management of obesity-induced asthma and the increasing prevalence of this condition has led to an urgent requirement for the development of an effective treatment^1,4^. We propose that empagliflozin and dulaglutide, alone and combined could be used to safely treat obesity-induced asthma. This new pharmacotherapy might contribute to reducing the medical cost associated with unresponsiveness to conventional therapies and improve the clinical efforts to treat obesity-induced asthma.

Obesity accompanies various comorbid diseases including hypertension, diabetes, cardiovascular disease, renal insufficiency, and even cancer, and it causes tremendous economic burden^29,30^. Empagliflozin and dulaglutide could improve metabolic abnormality, lead to weight loss, and contribute to treating other obesity-related comorbid diseases^31,32^. Empagliflozin has shown protective effects on the cardiovascular and renal system, similar to the effects of dulaglutide^33^. We believe that these drugs could be prescribed to asthma patients with obesity and could be helpful in controlling other obesity-related comorbidities.

This study has some limitations. First, the specific action mechanisms of empagliflozin and dulaglutide on the obesity-induced asthma were not revealed. We suggest numerous possible mechanisms; weight loss, restoration of metabolic abnormality, anti-inflammatory effects, and anti-fibrotic effects that may contribute to the treatment efficacy. However, further studies using inhibitors of specific cytokine or molecules are required to determine the detailed mechanisms. Second, a multimodal approach such as the combination of pharmacotherapy, reduction of dietary intake, or exercise or a combination of these activities may be more helpful to manage obesity-induced asthma than drug treatment alone, and further studies are needed to prove the hypothesis. Third, other effects of these drugs on the liver, heart, and kidney were not investigated and this should be a focus of future investigations. In addition, previous study showed obesity impairs macrophage and monocyte efferocytosis, and it reduces glucocorticoid responsiveness^34^. If improvement of glucocorticoid responsiveness after treatment of study drugs was observed, it would be interesting. Last, we used only male murine model, because we could not obtain sufficient weight gain to induced significant AHR and fibrosis using female murine model in preliminary model. Although there have been controversial, some clinical studies have revealed that female is more susceptible to obesity associated asthma than male^35^. We should carefully interpret results of this study based on them.

This is the first study to indicate that empagliflozin and dulaglutide could be promising candidates in the treatment of obesity-induced AHR and fibrosis, using a murine HFD model. Weight loss, restoration of metabolic abnormality, anti-inflammatory, and anti-fibrotic effects of these drugs may be involved in the mechanisms. Co-treatment with these two drugs had an additive effects and further randomized clinical trials may be needed to prove these findings.

## Methods

### Animals and study design

**Figure 5:**
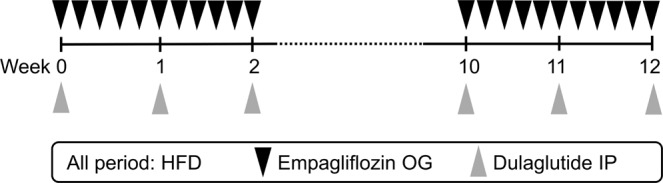
Scheme of study. HFD, high-fat diet; OG, oral gavage; IP, intraperitoneal.

We assigned mice to the following five groups (n = 5–7 per group): normal diet control (n = 6), HFD-induced obesity (n = 7), HFD/EMP (n = 5), HFD/DUL (n = 7), and HFD/combi (n = 7). The study scheme is shown in Fig. 5. All the mice were maintained in conventional animal facilities under standard conditions (room temperature, 21–24 °C; relative humidity, 45–70%; 12-h light/dark cycle), which has been fully accredited by the Association for Assessment and Accreditation of Laboratory Animal Care International. The 4-week-old male C57BL/6 mice (Japan-SLC, Hamamatsu, Japan) were fed an HFD (D12492; Research Diets, Inc., New Brunswick, USA; fat accounting for 60% of the calories) for 12 weeks to establish the HFD-induced obesity model. Lean mice in the control group were fed a normal chow diet (D12450B; fat accounting for 10%). Empagliflozin (Jardiance, 10 mg·kg^−1^·day^−1^, Boehringer Ingelheim, Germany) dissolved with 0.5% hydroxyethylcellulose (Sigma-Aldrich, Germany) or the solvent was administered by oral gavage five times per week. Dulaglutide (Trulicity, 0.6 mg·kg^−1^·week^−1^, Eli Lilly, IN, USA) dissolved with 0.9% saline or the solvent was administered intraperitoneally once a week. The HFD/combi group was co-administered empagliflozin (10 mg·kg^−1^·day^−1^) and a reduced dose of dulaglutide (0.3 mg·kg^−1^·week^−1^). In control group and HFD group, solvent of both two drugs was administered according to the above protocol. In HFD/EMP group, empagliflozin and solvent of dulaglutide were administered according to the above protocol. In HFD/DUL group, dulaglutide and solvent of empagliflozin were treated along with the above protocol. The dose in empagliflozin (10 mg·kg^−1^·day^−1)^ and dulaglutide (0.6 mg·kg^−1^·week^−1^) were selected based on the previous studies^36,37^, and the combination dose of dulaglutide (0.6 mg·kg^−1^·week^−1^) was chosen based on our preliminary studies. The study protocol was approved by the Institutional Animal Care and Use Committee of the Yonsei University College of Medicine (Seoul, Korea), which has been fully accredited by the Association for Assessment and Accreditation of Laboratory Animal Care International, and conducted according to the relevant guidelines and regulations of the institution.

### Fasting glucose level, GTT, serum levels of leptin and insulin

The fasting glucose level and oral GTT were performed at baseline week 0 and final week 12. Mice were starved overnight for the fasting glucose level analysis and GTT. Fasting glucose was measured before the start of GTT. The GTT was performed after orally administering glucose (2 g/kg body weight; Sigma-Aldrich, St. Louis, MO, USA). Blood from the tail vein was measured at 0, 30, 60, 90, and 120 minutes after glucose injection to determine glucose level using the Accu-Check glucometer (Roche, Mannheim, Germany). Serum levels of leptin (R&D system, Inc., Minneapolis, MN) and insulin (Alpc, Salem, NH, USA) on the final date were measured using commercial ELISA kit according to the suggested protocol, respectively.

### Measurement of methacholine AHR

The mice were anesthetized with pentobarbital sodium (50 mg/kg; Hanlim Pharma Co., Seoul, Korea) by intraperitoneal injection 48 h after the last challenge. An 18-gauge cannula was inserted into each anesthetized mouse via tracheostomy, and then the mouse was connected to a ventilator. AHR in response to various concentrations of inhaled aerosolized methacholine (MCh, 6.25, 12.5, 25.0, 50.0, and 100.0 mg/mL; Sigma-Aldrich) was measured using a forced oscillation technique (FlexiVent® 5.1; SCIREQ, Montreal, Canada) as previously described^38^.

### Collection and processing of BALF

To collect BALF, the mouse lungs were irrigated with 1 mL Hank’s balanced salt solution (HBSS, Thermo Fisher Scientific, Waltham, MA, USA) through the tracheal tube and the total number of cells was counted using a haemocytometer. The collected BALF samples were centrifuged for 3 min at 10,000 rpm and 4 °C and the supernatants were stored at −80 °C. The whole cell pellets were resuspended in HBSS, and BALF cell smears were prepared by cytocentrifugation (Cytospin 3, Thermo, Billerica, MA, USA). The cytocentrifuged slides were stained with Leukostat (Fisher Diagnostics, Fair Lawn, NJ, USA) to count at least 200 inflammatory cells as previously described^38^.

### Histological analysis

The left lungs of the mice were fixed in 4% formalin and embedded in paraffin. Lung sections were cut and stained with H&E for general examination, PAS to measure goblet cell hyperplasia, and MT to assess fibrosis as previously described^39^. Tissue sections were examined using an Olympus BX40 microscope in conjunction with an Olympus U-TV0.63XC digital camera (Olympus BX53F, Center Valley, PA, USA). Images were acquired using the CellSens standard 1.6 image software. Fibrosis was quantitatively analysed using Metamorph® (Molecular Devices, Sunnyvale, CA, USA). The fibrotic area was assessed by measuring the colour-pixel count over the pre-set threshold colour for the entire field containing several bronchial tubes on MT-stained slides at 200× magnification. In addition, a commercially available hydroxyproline assay kit (Cell Biolabs, Inc., San Diego, USA) was used to quantify fibrosis in lung homogenates.

### Enzyme-linked immunosorbent assay

To analyse cytokine levels, the right lung tissues were homogenized with 50 mg/mL tissue protein extraction buffer (ThermoFisher Scientific Inc., Rockford, IL, USA) using a tissue homogenizer (Biospec Products, Bartlesville, OK, USA). After incubation for 30 min on ice, homogenates were centrifuged at 14,000 rpm for 10 min. Supernatants of lung homogenates were collected and stored at −80°C for measurement of cytokine levels. Concentrations of IL-17, TGF-*β*1, IL-1*β*, TNF-*α*, and IL-6 in lung homogenate were measured using an enzyme-linked immunosorbent assay (ELISA, R&D Systems, San Diego, CA, USA) according to the manufacturer’s instructions.

### RNA extraction and real-time polymerase chain reaction (PCR)

Total RNA was extracted from lung tissues using in TRIzol® reagent (Ambion, Life technologies, Carlsbad, CA, USA) by homogenization using a tissue homogenizer (T10 basic ULTRA-TURRAX®, IKA, Staufen, Germany) according to the manufacturer’s instructions. Reverse transcription was performed using reverse transcriptase (Invitrogen, Carlsbad, CA, USA) primed with oligo (dT) primer. The synthesized cDNAs were amplified using the SYBR® green PCR master mix (BioRad, California, USA) and forward and reverse primers (Bioneer, Daejeon, Korea) using a real-time PCR system (StepOnePlus, Applied Biosystems, Foster City, CA, USA). All the PCR experiments were performed under the following conditions: 95 °C for 5 min, 95 °C for 15 s, and 60 °C for 45 s for up to 40 cycles.

### Flow cytometry

Cells obtained from the lung tissues were stained with fluorescent dye-conjugated CD3 (T-cell marker), CD4 (T helper cell marker), C-X-C motif chemokine receptor 3 (CXCR3, Th1 cell marker), C-C motif chemokine receptor 4 (CCR4, Th2 cell marker), CCR6 (Th17 cell marker), and CD8 (T cytotoxic cell marker) antibodies (eBiosciences, San Diego, CA, USA). Stained cells were measured using a BD FACSVerse™ flow cytometer and analysed using the FlowJo 8.3.3 software (BD Bioscience, San Jose, CA, USA).

### Statistical analysis

All results are expressed as the mean ± standard error of mean. The weight change and AHR data among the groups were analysed using a repeated-measures analysis of variance (ANOVA) followed by a post-hoc Bonferroni test. One-way ANOVA followed by a post-hoc Bonferroni test was performed to compare the numeric variables among groups. The GTT-AUC was obtained as the calculated AUC of glucose level changes according to the time after GTT. We used the statistical package for the social sciences (SPSS) software version 12.0 (SPSS Inc., Chicago, IL, USA) for the analyses and a *P* < 0.05 was considered statistically significant.

## Data Availability

The datasets generated during and/or analysed during the current study are available from the corresponding author on reasonable request.
